# Alters- und Geschlechteraspekte bei der Inanspruchnahme von Notaufnahmen und ihre Berücksichtigung in der Steuerung der Notfallversorgung

**DOI:** 10.1007/s00103-026-04252-4

**Published:** 2026-06-10

**Authors:** Kalina Witt, Viktoria Bogner-Flatz, Ulrike von Arnim, Mandy Schulz, Kristine Huber, Claudia Ritter-Rupp, Sarah Oslislo

**Affiliations:** 1 Zentralinstitut für die kassenärztliche Versorgung in der Bundesrepublik Deutschland, Salzufer 8, 10587 Berlin, Deutschland; 2 Kassenärztliche Vereinigung Bayerns, München, Deutschland; 3 Zentrale Notaufnahme, Klinikum Ebersberg München Ost, Ebersberg, Deutschland; 4 Zentrale Notaufnahme, Vivantes Netzwerk für Gesundheit GmbH, Klinikum Neukölln, Berlin, Deutschland; 5 Zentrum für klinische Notfall- und Akutmedizin, Standort Aschaffenburg, Klinikum Aschaffenburg-Alzenau, Aschaffenburg, Deutschland

**Keywords:** Patientenperspektive, Digitale Selbsteinschätzung, Geschlechterunterschiede, Altersspezifische Unterschiede, Bekanntheit 116117, Patient perspective, Digital self-assessment, Gender differences, Age-specific differences, Awareness 116117

## Abstract

**Einleitung:**

Die Versorgung in Notaufnahmen sollte patienten- und bedarfsgerecht gesteuert werden. Alter und Geschlecht beeinflussen Konsultations- und Versorgungsmuster. Ziel der Studie ist die Analyse dieser Faktoren sowie vorgelagerter Versorgungspfade zur Reduktion von Fehlsteuerungen und Verbesserung der Versorgungsqualität.

**Methoden:**

Zwischen März und November 2024 wurden standardisierte Patientenbefragungen in 5 Berliner und 18 bayerischen Notaufnahmen durchgeführt (*n* = 10.040). Erfasst wurden Soziodemografie, Beschwerden, subjektive Dringlichkeit, Arztkontakte und die Bekanntheit der 116117. Einflussfaktoren wurden mittels logistischer Regression analysiert; berücksichtigt wurden nur Fälle mit Angabe „männlich“ oder „weiblich“.

**Ergebnisse:**

Insgesamt konnten 9835 Fragebögen ausgewertet werden (49,6 % Frauen); die Patient:innen gehörten überwiegend älteren Altersgruppen an. Die Vorstellungsgründe unterschieden sich nach Geschlecht: Frauen nannten häufiger Schmerzen sowie neurologische oder abdominale Beschwerden, Männer eher Verletzungen. Gesundheitsbezogene Ängste waren für rund 37,8 % ein zentrales Inanspruchnahmemotiv, häufiger bei Frauen. Einen vorherigen Kontaktversuch zur vertragsärztlichen Versorgung gaben 48,7 % der Frauen und 44,2 % der Männer an (*p* < 0,001). Direkte Notaufnahmebesuche waren häufiger bei höherer Dringlichkeit und Verletzungen. Die Bekanntheit der 116117 lag bei 67,0 % der Frauen und 60,3 % der Männer und nahm mit dem Alter ab; die Bereitschaft zur digitalen Selbsteinschätzung war bei Jüngeren höher.

**Diskussion:**

Alter und Geschlecht beeinflussen Inanspruchnahme und Steuerbarkeit der Notfallversorgung. Zielgruppensensible, niedrigschwellige und digitale Angebote können Fehlsteuerungen reduzieren und die Versorgungsqualität verbessern.

**Zusatzmaterial online:**

Zusätzliche Informationen sind in der Online-Version dieses Artikels (https://doi.org/10.1007/s00103-026-04252-4) enthalten.

## Abstract (en)

**Introduction:**

Emergency department (ED) care should be guided according to patient needs and demand. Age and gender influence consultation and care patterns. This study aims to analyze these factors as well as upstream care pathways in order to reduce misallocation and improve quality of care.

**Methods:**

Between March and November 2024, standardized patient surveys were conducted in 5 Berlin and 18 Bavarian emergency departments (*n* = 10,040). Data collected included sociodemographic characteristics, presenting complaints, subjective urgency, physician contacts, and awareness of the 116117 hotline. Influencing factors were analyzed using logistic regression; only cases indicating “male” or “female” were included.

**Results:**

A total of 9835 questionnaires were analyzed (49.6% female); patients were predominantly from older age groups. Reasons for presentation differed by gender: for example, women more frequently reported pain as well as neurological or abdominal complaints, whereas men more often presented with injuries. Health-related anxiety was a key motive for seeking care in about 37.8% of cases and was more common among women. A prior attempt to contact ambulatory care was reported by 48.7% of women and 44.2% of men (*p* < 0.001). Direct ED visits were more frequent in cases of higher urgency and injuries. Awareness of the 116117 hotline was 67.0% among women and 60.3% among men and decreased with age; willingness to use digital self-assessment tools was higher among younger individuals.

**Discussion:**

Age and gender influence both utilization patterns and the steerability of emergency care. Target group-specific, low-threshold, and digital interventions may help reduce misallocation and improve quality of care.

## Einleitung

Die Notaufnahmepopulation weist eine ausgeprägte Heterogenität auf, die sich insbesondere in den Dimensionen Alter und klinische Präsentation manifestiert [1–3]. Diese Faktoren beeinflussen die Inanspruchnahme der Notaufnahme, die Wahrnehmung von Dringlichkeit sowie zentrale Prozess- und Ergebnisparameter der Notfallversorgung. Dadurch prägen sie – oftmals implizit – Versorgungspfade, klinische Abläufe und Versorgungsoutcomes, auch jenseits objektiver medizinischer Dringlichkeit [2–4]. Mit dem demografischen Wandel steigt der Anteil älterer Patient:innen – etwa 40 % der erwachsenen Notfallpatient:innen sind derzeit ≥ 65 Jahre alt [1]. Auch das Geschlecht spielt eine relevante Rolle. Frauen zeigen oftmals andere Behandlungsverläufe und schlechtere subjektive Versorgungserfahrungen [5–7].

Alter und Geschlecht sind mit unterschiedlichen Nutzungsmustern und Versorgungserfahrungen in der Notfallversorgung assoziiert [3, 8, 9]. Frauen erleben häufiger Verzögerungen, Bias in Diagnostik und Schmerzmanagement, während Männer höhere Mortalitäts- und Hospitalisierungsraten aufweisen [5]. Der Einfluss kombinierter Effekte von Alter und Geschlecht, wie unterschiedliche Behandlungspräferenzen, selbst definierte Therapieziele oder spezifische Priorisierungen, die insbesondere bei älteren Frauen beobachtet werden, ist bislang unzureichend erforscht [8, 10, 11]. Ein vertieftes Verständnis dieser Wechselwirkungen ist jedoch essenziell, um die Notfallversorgung patientenzentriert und bedarfsgerecht zu gestalten. Die vorhandene Evidenz unterstreicht die Notwendigkeit, die Alters- und Geschlechterperspektive systematisch in Versorgungspfade und Steuerungsprozesse der Notfallmedizin zu integrieren [11, 12]. Nur so lassen sich Versorgungsqualität und Effizienz verbessern sowie Fehlsteuerungen und ungleiche Behandlung verhindern.

Ziel der vorliegenden Studie war es, geschlechterspezifische und altersabhängige Unterschiede in der Inanspruchnahme von Notaufnahmen sowie der vorgelagerten Versorgungspfade zu identifizieren.

## Methoden

Die Studie wird nach *The Strengthening the Reporting of Observational Studies in Epidemiology* (STROBE) berichtet [12]. Zur Untersuchung patientenseitiger Einflussfaktoren auf die Notaufnahmeinanspruchnahme wurden 2024 2 standardisierte, querschnittliche Patientenbefragungen [13, 14] in Anlehnung an Somasundaram et al. (2018) [15] durchgeführt.

### Studiendesign und Datenerhebung

Die erste Erhebung fand vom 18.03. bis 18.04.2024 in 5 Berliner [13], die zweite vom 03.09. bis 29.11.2024 in 18 bayerischen Notaufnahmen [14] statt. Die Rekrutierung von Patient:innen erfolgte in Warte- und Behandlungsbereichen der Notaufnahmen an allen Wochentagen von 10.00 bis 20.00 Uhr bzw. 11.00 bis 21.00 Uhr. Patient:innen wurden durch geschulte Mitarbeitende aktiv angesprochen, sobald es der klinische Ablauf zuließ und keine akute Behandlungspriorität bestand. Die Ansprache erfolgte in Abhängigkeit von Personalverfügbarkeit, Patientenaufkommen und klinischer Situation. Aufgrund zeitlich begrenzter Rekrutierungsfenster, hoher klinischer Auslastung sowie organisatorischer und personeller Limitationen konnten nicht alle potenziell geeigneten Patient:innen aktiv angesprochen werden.

Die Beantwortung eines Onlinefragebogens war mittels Tablets oder des eigenen Smartphones möglich. Die Bearbeitung der Fragebögen konnte selbstständig oder mit Unterstützung durch Mitarbeitende erfolgen. Die Befragungen wurden anonym durchgeführt; es wurden keine personenbezogenen Daten erhoben und keine Verknüpfung mit Behandlungs- oder Routinedaten vorgenommen.

### Fragebogen und erhobene Merkmale

Der Fragebogen wurde für die Berliner Befragung entwickelt und für die bayerische Erhebung leicht angepasst. Er umfasste 19 Haupt- und bis zu 8 Zusatzfragen zu folgenden Merkmalen aus 5 Kernbereichen:

- *Soziodemografie:* Alter (kategoriale Gruppierung), Geschlecht, Bildungsstand nach International Standard Classification of Education (ISCED; [16]), Migrationshintergrund [17];
- *medizinische Merkmale:* Hauptsymptome nach Canadian Emergency Department Information System (CEDIS; [18]); Schmerzintensität (numerische Rating-Skala (NRS) 1–10; [19]), Beschwerdedauer (6 Kategorien) und -entwicklung (akut, schleichend, konstant, nachlassend). Beim medizinischen Vorstellungsgrund konnten bei Bedarf mehrere zutreffende Beschwerden ausgewählt werden (Mehrfachauswahl).
- *Nutzungsprofile:* Zugangswege zur Notaufnahme (Selbstvorstellung, Rettungsdienst/Krankentransport), vorheriger vertragsärztlicher Kontaktversuch;
- *Inanspruchnahmemotive:* subjektive Dringlichkeit und Inanspruchnahmemotive. Die subjektive Dringlichkeit wurde mittels einer Selbsteinschätzung erhoben. Die Personen wurden gefragt: „Wie dringend schätzen Sie Ihre aktuellen Beschwerden ein?“ Die Antwort erfolgte auf einer 5‑stufigen Likert-Skala („nicht dringend“, „wenig dringend“, „dringend“, „sehr dringend“, „Notfall“). Für die statistischen Analysen wurde die Variable ordinal verwendet.
- *Patientensteuerung:* Bekanntheit der Rufnummer des Patientenservices der Kassenärztlichen Vereinigungen (KV; 116117). Die Bereitschaft zur Nutzung digitaler Selbsteinschätzungsangebote (wie z. B. DocYet, strukturierte medizinische Ersteinschätzung in Deutschland (SmED) als Patientennavi über die 116117.de, Ada, Symptoma) wurde laienverständlich formuliert und ohne Fachterminologie mit der Frage: „Würden Sie Ihre Beschwerden mittels einer digitalen Anwendung online einschätzen?“, erhoben.

Zusätzlich wurde erhoben, ob sich Patient:innen vor dem Notaufnahmebesuch online über ihre Beschwerden oder geeignete Versorgungspfade informiert hatten.

Eine detaillierte Übersicht über alle erhobenen Variablen, die exakten Frageformulierungen sowie die jeweiligen Antwortkategorien ist von Witt et al. (2024) veröffentlicht worden [14].

Für die Befragung wurde eine Stichprobengröße von *n* = 10.000 angestrebt, welche sich in erster Linie an Machbarkeitsüberlegungen und bisherigen Rekrutierungserfahrungen aus der Befragung von Somasundaram et al. (2018) [15] orientiert. Die angestrebte Stichprobengröße von etwa 500 Teilnehmenden pro Notaufnahme wurde auf Grundlage einer konservativen Prävalenzschätzung von 50 % berechnet. Dies entspricht einem 95 %-Konfidenzintervall mit einer Genauigkeit von ±2 % bei einem Standardfehler von 2 %. Zusätzlich wurde eine Ausfallquote von 20 % in die Berechnung einbezogen.

### Einschluss- und Ausschlusskriterien

Im Rahmen des Screenings wurden alle aktiv ansprechbaren Patient:innen anhand der nachfolgenden Ein- und Ausschlusskriterien geprüft. Eingeschlossen wurden erwachsene Patient:innen (sowie von Erziehungsberechtigten begleitete Minderjährige), sofern kein sofortiger Behandlungsbedarf (MTS rot/ESI 1)^1^, keine Einwilligungsunfähigkeit und ausreichende Deutschkenntnisse vorlagen. Ausgeschlossen wurden Patient:innen mit unmittelbarem Behandlungsbedarf, eingeschränkter Einwilligungsfähigkeit, fehlenden Deutschkenntnissen sowie Patient:innen, die aufgrund der klinischen Situation oder aus organisatorischen Gründen nicht angesprochen werden konnten.

### Statistische Auswertung

Zur Beschreibung der Studienpopulation wurden die Antwortoptionen „Weiß nicht/Keine Angabe“ als fehlende Werte klassifiziert und von der weiteren Analyse ausgeschlossen.

Bei den geschlechtsspezifischen Auswertungen konnte die Kategorie „divers“ (*n* = 11) nicht berücksichtigt werden, da für diese Gruppe aufgrund der geringen Fallzahl keine hinreichend belastbaren statistischen Analysen möglich waren. Die medizinischen Vorstellungsgründe wurden für die statistischen Analysen jeweils dichotom (vorhanden vs. nicht vorhanden) berücksichtigt. Urologische und gynäkologische Beschwerden wurden ausschließlich deskriptiv dargestellt. Da der Fragebogen überwiegend optional zu beantwortende Items enthielt, variiert die Stichprobengröße je nach Variable; das jeweilige *n* wird bei den entsprechenden Auswertungen ausgewiesen.

Bezogen auf die Endpunkte „Motive der Inanspruchnahme“, „vorheriger Kontaktversuch zur vertragsärztlichen Versorgung“, „Bekanntheit Patientenservice 116117“ und „Bereitschaft zur Nutzung digitaler Angebote“ wurden im Rahmen von deskriptiver Statistik Patientenzahlen bzw. prozentuale Anteile mit entsprechender Ausprägung der Variablen berichtet. Gruppenvergleiche wurden mittels Chi-Quadrat-Tests durchgeführt.

Der Endpunkt „vorheriger vertragsärztlicher Kontaktversuch“ wurde dichotom operationalisiert (Ja/Nein). Als vorheriger Kontaktversuch galt jede dokumentierte Kontaktaufnahme zu einer vertragsärztlichen Versorgung (z. B. Hausarztpraxis, Facharztpraxis, Notdienstpraxis oder Ärztlicher Bereitschaftsdienst über die 116117) im Vorfeld der aktuellen Notaufnahmeinanspruchnahme, unabhängig davon, ob diese erfolgreich war.

Zur Analyse der Einflussfaktoren auf einen vorherigen Kontaktversuch zur vertragsärztlichen Versorgung wurde aufgrund vielfältiger geschlechtsspezifischer Ausprägungen der Schätzer jeweils ein logistisches Regressionsmodell für weibliche und männliche Befragte berechnet, in welches soziodemografische Variablen, subjektive Dringlichkeit, Schmerzintensität und medizinische Vorstellungsgründe einbezogen wurden. Kinder und Jugendliche unter 18 Jahren wurden in die deskriptiven Auswertungen einbezogen, da sie einen relevanten Anteil der Notaufnahmepopulation darstellen und altersübergreifende Vergleiche der Inanspruchnahmemuster somit ermöglicht werden. Aus den multivariablen Regressionsanalysen wurden sie jedoch ausgeschlossen, da die pädiatrische Teilpopulation aufgrund der Versorgungsstrukturen der beteiligten Notaufnahmen selektiv sein kann. Das Alter wurde als kategoriale Variable modelliert (< 18 Jahre sowie erwachsene Altersgruppen in Dekaden), um nichtlineare altersabhängige Unterschiede im vorherigen vertragsärztlichen Kontaktversuch abzubilden.

Die Auswertung erfolgte mit IBM SPSS Statistics (Version 20) und R Studio (Version 2023.12.1).

## Ergebnisse

**Abb. 1:**
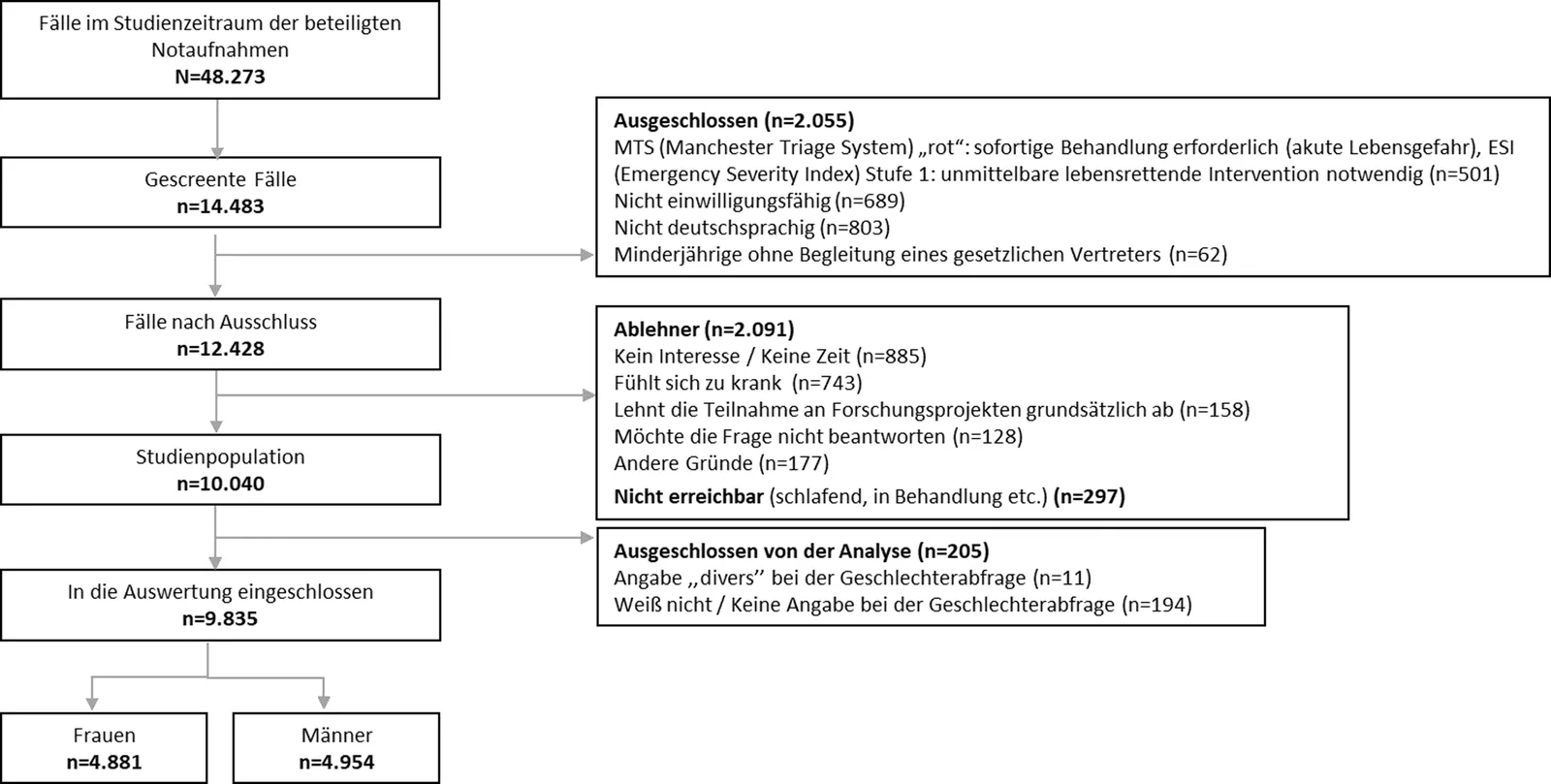
Rekrutierungs- und Einschlussprozess

Insgesamt suchten im gesamten Erhebungszeitraum *N* = 48.273 Patient:innen die beteiligten Notaufnahmen auf. Diese Zahl umfasst sämtliche Konsultationen während des Studienzeitraums, unabhängig von Tageszeit oder Wochentag. Davon wurden *n* = 14.483 Patient:innen systematisch hinsichtlich der Einschluss- und Ausschlusskriterien gescreent (Abb. 1). Von diesen erfüllten *n* = 12.428 Patient:innen die Einschlusskriterien. Von diesen lehnten 2091 die Teilnahme ab oder waren nicht erreichbar (*n* = 297), sodass insgesamt 10.040 Notaufnahmepatient:innen an der Befragung teilnahmen. Nach Ausschluss von Fragebögen, bei denen Angaben zum Geschlecht fehlten (*n* = 194) oder „divers“ angegeben wurde (*n* = 11), verblieben 9835 auswertbare Fragebögen. Der Frauenanteil lag bei 49,6 %.

### Charakteristika und medizinische Vorstellungsgründe der Teilnehmenden

Es zeigten sich signifikante geschlechtsspezifische Unterschiede in der Alters- und Bildungsstruktur (jeweils *p* < 0,001): Männer waren etwas häufiger unter 18 Jahre sowie zwischen 50 und 69 Jahre alt und wiesen öfter einen hohen Bildungsstand auf, während Frauen überproportional häufig ≥ 70 bzw. ≥ 80 Jahre alt waren (Tab. 1). Hinsichtlich des Migrationshintergrunds und der selbstständigen Vorstellung in der Notaufnahme (72,7 %) bestanden keine Unterschiede zwischen den Geschlechtern.

**Tab. 1:** Charakteristika der befragten Patient:innen nach Geschlecht

	Weiblich *n* = 4881		Männlich *n* = 4954		
	Anzahl	Anteil in %	Anzahl	Anteil in %	*p*-Wert
**Altersgruppen ***(n* *=* *9818)*	4872	49,6	4946	50,4	–
Unter 18 Jahre	275	5,6	394	8,0	*<* *0,001*
18–29 Jahre	732	15,0	686	13,9	
30–39 Jahre	633	13,0	664	13,4	
40–49 Jahre	564	11,6	630	12,7	
50–59 Jahre	622	12,8	725	14,7	
60–69 Jahre	634	13,0	716	14,5	
70–79 Jahre	650	13,3	573	11,6	
Älter als 80 Jahre	762	15,6	558	11,3	
**Bildungsstand nach ISCED** *(n* *=* *8929)*	4412	49,4	4517	50,6	–
Niedrig	641	14,5	546	12,1	*<0,001*
Mittel	2391	54,2	2174	48,1	
Hoch	1380	31,3	1797	39,8	
**Migration** *(n* *=* *9719)*	4826	49,7	4893	50,3	–
Kein Migrationshintergrund	3732	77,30	3742	76,50	0,324
Migrationshintergrund	1094	22,70	1151	23,50	
**Zugangsweg ***(n* *=* *9632)*	4773	49,6	4859	50,4	–
Selbstständig	3462	72,5	3547	73,0	0,615
Rettungsdienst/Krankentransport	1331	27,5	1312	27,0	
**Medizinischer Vorstellungsgrund ***(n* *=* *9835; Mehrfachantworten)*					
Allgemeine Beschwerden	332	6,8	309	6,2	0,270
Schmerzen	1434	29,4	1340	27,0	*0,011*
Verletzung	1084	22,2	1405	28,4	*<* *0,001*
Herz-Kreislauf Beschwerden/Atembeschwerden	764	15,7	845	17,1	0,060
Bauchbeschwerden	566	11,6	456	9,2	*<* *0,001*
Neurologische Beschwerden/psychiatrische Beschwerden	464	9,5	321	6,5	*<* *0,001*
Beschwerden auf der Haut	115	2,4	127	2,6	0,516
Beschwerden im Hals-Nasen-Ohren-Bereich	175	3,6	180	3,6	0,914
Urologische Beschwerden	131	2,7	237	4,8	–
Gynäkologische Beschwerden	163	3,3	1	0,0	–
Augenprobleme	116	2,4	102	2,1	0,304
**NRS-Schmerzabfrage ***(n* *=* *6658)*	3345	50,2	3313	49,8	–
Gering (1–3)	403	12,0	584	17,6	*<* *0,001*
Mittel (4–6)	1175	35,1	1196	36,1	
Stark (7–10)	1767	52,8	1533	46,3	
**Subjektive Dringlichkeit ***(n* *=* *9554)*	4741	49,6	4813	50,4	–
Nicht dringlich	60	1,3	74	1,5	0,099
Weniger dringlich	487	10,3	546	11,3	
Dringlich	1942	41,0	1988	41,3	
Sehr dringlich	1470	31,0	1402	29,1	
Notfall	782	16,5	802	16,7	

*ISCED* International Standard Classification of Education, *NRS* numerische Rating-Skala

Männer berichteten als medizinischen Vorstellungsgrund häufiger Verletzungen, Frauen dagegen häufiger Schmerzen (insbesondere starke Schmerzen NRS 7–10), Bauch- sowie neurologische bzw. psychiatrische Beschwerden. Die subjektive Dringlichkeit wurde von beiden Geschlechtern überwiegend als (sehr) dringlich eingeschätzt.

Weitere Charakteristika der befragten Patient:innen nach Altersgruppen sind im Onlinematerial zu finden.

### Motive der Inanspruchnahme

**Abb. 2:**
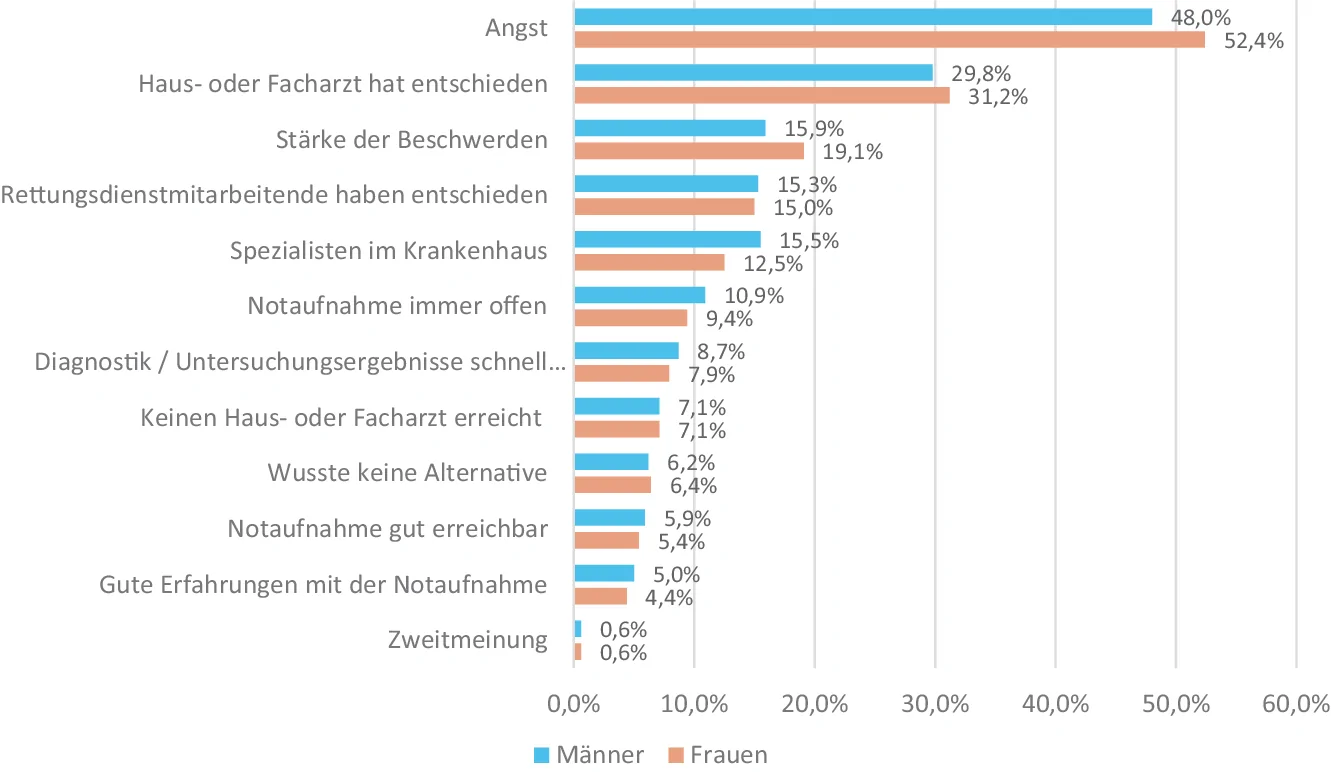
Motive der Inanspruchnahme nach Geschlecht (*n* = 7402), Mehrfachantworten waren möglich

Bei den Inanspruchnahmemotiven gaben 3714 (37,8 %) der Befragten an, die Notaufnahme aufgrund von gesundheitsbezogenen Ängsten aufgesucht zu haben. Dieses Motiv war bei Frauen signifikant häufiger als bei Männern (*p* < 0,001; Abb. 2). Männer suchten signifikant häufiger die Notaufnahme aufgrund der dort erhofften Behandlung durch Spezialisten auf (*p* < 0,001). Weitere Motive und ihre geschlechtsspezifische Ausprägung sind der Abb. 2 zu entnehmen.

Von den Befragten ab 50 Jahren (*n* = 5240) gaben 51,4 % an, die Notaufnahme auf Empfehlung eines Haus- oder Facharztes aufgesucht zu haben (*p* < 0,001), während Befragte unter 50 Jahren häufiger angaben, die Notaufnahme aufgrund der Nichterreichbarkeit eines niedergelassenen Arztes aufgesucht zu haben (*p* < 0,001).

### Vorheriger Kontaktversuch zur vertragsärztlichen Versorgung

48,7 % der weiblichen und 44,2 % der männlichen Befragten hatten vorab einen Kontaktversuch zur vertragsärztlichen Versorgung (*n* = 9835; *p* < 0,001). Von den Befragten ab 50 Jahren (*n* = 5240) gaben 48,7 % einen vorherigen Kontaktversuch an, häufiger als jüngere Befragte (*p* < 0,001).

In den geschlechterspezifischen multivariablen logistischen Regressionsanalysen wurde die Wahrscheinlichkeit einer direkten Inanspruchnahme der Notaufnahme ohne vorherigen vertragsärztlichen Kontaktversuch modelliert. Verletzungen und eine höhere subjektive Dringlichkeit waren bei beiden Geschlechtern signifikant mit einer erhöhten Wahrscheinlichkeit einer direkten Notaufnahmeinanspruchnahme assoziiert. Demgegenüber gingen Bauch- und Hautbeschwerden häufiger mit einem vorherigen vertragsärztlichen Kontaktversuch einher. Geschlechterspezifische Unterschiede zeigten sich bei neurologischen Beschwerden (signifikant nur bei Frauen), HNO-Beschwerden (signifikant nur bei Männern). Beide waren mit einer höheren Wahrscheinlichkeit eines vorherigen Kontaktversuchs assoziiert. Zudem waren Schmerzen als Hauptanlass bei Frauen sowie eine mittlere Schmerzintensität bei Männern mit einer höheren Wahrscheinlichkeit eines vertragsärztlichen Kontaktversuchs verbunden.

**Tab. 2:** Logistische Regressionsanalysen zur direkten Inanspruchnahme der Notaufnahme ohne vorherigen vertragsärztlichen Kontaktversuch

	Frauen *n* = 2797					Männer *n* = 2729				
Variable	Odds Ratio	95 % Konfidenzintervall			*p-*Wert	Odds Ratio	95 % Konfidenzintervall			*p*-Wert
*Altersgruppen (REF 18–29 Jahre)*										
30–39 Jahre	0,963	0,733	1,263	0,783		0,984	0,743	1,305		0,913
40–49 Jahre	1,093	0,825	1,448	0,534		0,901	0,677	1,201		0,478
50–59 Jahre	0,989	0,748	1,308	0,941		1,006	0,757	1,336		0,97
60–69 Jahre	0,908	0,682	1,208	0,506		0,808	0,602	1,083		0,154
70–79 Jahre	1,009	0,746	1,365	0,953		0,678	0,494	0,932		*0,016*
Älter als 80 Jahre	1,503	1,123	2,014	*0,006*		0,669	0,476	0,939		*0,02*
*Bildungsstand nach ISCED (REF ISCED niedrig)*										
ISCED mittel	0,835	0,77	1,272	0,933		0,783	0,581		1,056	0,109
ISCED hoch	1,099	0,638	1,092	0,187		0,675	0,497		0,918	*0,012*
*Migrationshintergrund (REF kein Migrationshintergrund)*										
Migrationshintergrund	1,099	0,914	1,322	0,314		1,098	0,909		1,326	0,332
*Subjektive Dringlichkeit*										
Subjektive Dringlichkeit	1,167	1,062	1,281	*0,001*		1,112	1,024		1,231	*0,014*
*Medizinischer Vorstellungsgrund*										
Allgemeine Beschwerden	0,866	0,579	1,296	0,483		0,915	0,605		1,386	0,676
Schmerzen	0,804	0,675	0,959	*0,015*		1,024	0,851		1,232	0,8
Verletzung	2,691	2,166	3,343	*<* *0,001*		2,854	2,292		3,553	*<* *0,001*
Herz-Kreislauf- und Atembeschwerden	0,983	0,752	1,285	0,901		1,221	0,942		1,583	0,131
Bauchbeschwerden	0,637	0,497	0,816	*<* *0,001*		0,685	0,524		0,897	*0,006*
Neurologische Beschwerden/psychiatrische Beschwerden	0,644	0,476	0,871	*0,004*		0,77	0,517		1,148	0,2
Beschwerden auf der Haut	0,527	0,297	0,936	*0,029*		0,519	0,314		0,857	*0,01*
Beschwerden im Hals-Nasen-Ohren-Bereich	0,767	0,516	1,142	0,192		0,66	0,438		0,994	*0,047*
Urologische Beschwerden	0,682	0,438	1,062	0,091		0,909	0,638		1,296	0,599
Gynäkologische Beschwerden	0,742	0,479	1,148	0,18		–	–		–	–
Augenprobleme	0,899	0,519	1,555	0,703		0,966	0,533		1,75	0,909
*Schmerzintensität (REF gering)*										
Schmerz mittel	0,865	0,665	1,125	0,278		0,729	0,577		0,922	*0,008*
Schmerz stark	0,902	0,692	1,175	0,445		0,813	0,641		1,03	0,087

*REF* Referenzwert, *ISCED* International Standard Classification of Education

Ein hoher Bildungsstand war ausschließlich bei Männern mit einer höheren Wahrscheinlichkeit eines vorherigen Kontaktversuchs assoziiert. Während Frauen im Alter von über 80 Jahren eine höhere Wahrscheinlichkeit aufwiesen, die Notaufnahme ohne vorherigen vertragsärztlichen Kontaktversuch aufzusuchen, war bei Männern in den Altersgruppen 70–79 Jahre sowie über 80 Jahren eine geringere Wahrscheinlichkeit einer direkten Notaufnahmeinanspruchnahme zu beobachten. Die vollständigen Modellparameter sind Tab. 2 zu entnehmen.

### Bekanntheit des Patientenservices 116117 und Nutzung digitaler Angebote

**Abb. 3:**
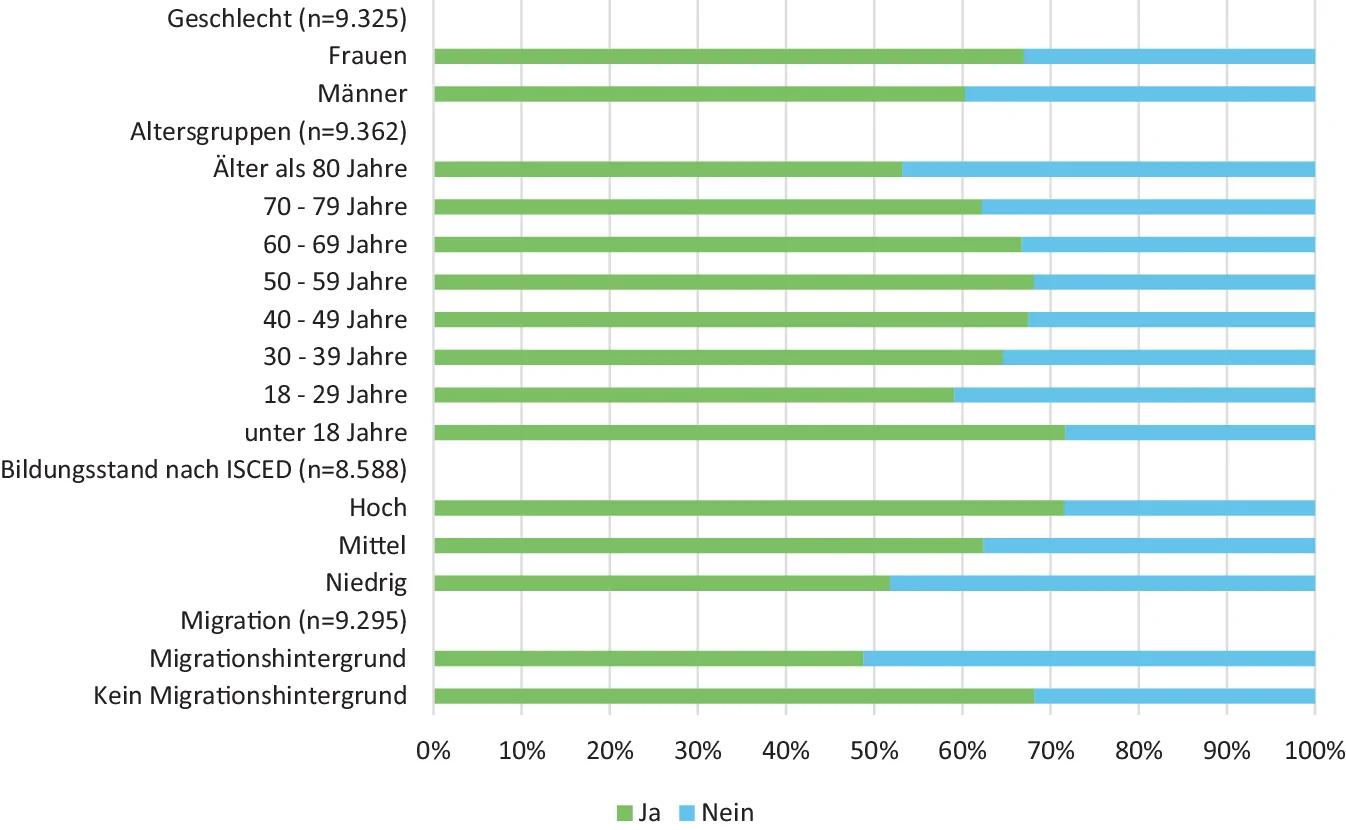
Bekanntheit der Rufnummer des Patientenservices 116117 der Kassenärztlichen Vereinigungen nach Geschlecht, Altersgruppen, Bildungsstand nach International Standard Classification of Education (ISCED) und Migration

67,0 % der weiblichen Befragten kannten die Rufnummer des Patientenservices (116117), bei den männlichen Befragten lag die Bekanntheit bei 60,3 % (*n* = 9325; *p* < 0,001). Die Verteilung der Bekanntheit nach soziodemografischen Merkmalen ist in Abb. 3 dargestellt.

**Abb. 4:**
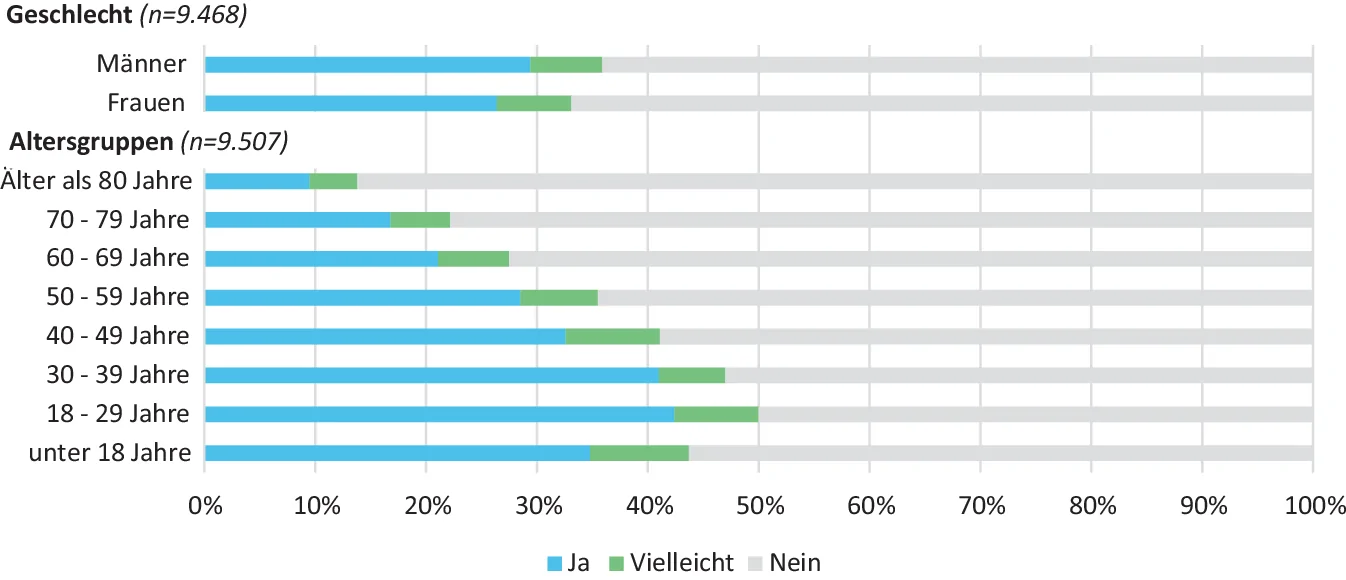
Bereitschaft zur Nutzung digitalen Selbsteinschätzung nach Geschlecht und Alter

31,9 % der 18- bis 29-jährigen Befragten hatten sich vor der Inanspruchnahme online über ihre Beschwerden informiert. Mit zunehmendem Alter nahm diese Nutzung ab (*n* = 9598; *p* < 0,001). Befragte ab 40 Jahren gaben zudem eine signifikant geringere Bereitschaft zur Nutzung digitaler Selbsteinschätzungsangebote an (*n* = 9507; *p* < 0,001; Abb. 4). Männer gaben insgesamt eine etwas höhere Bereitschaft zur Nutzung an als Frauen (29,4 % Männer vs. 26,4 % Frauen; *p* = 0,002).

## Diskussion

Die vorliegende Studie untersucht die Bedeutung von Alter und Geschlecht für das Inanspruchnahmeverhalten in deutschen Notaufnahmen und liefert damit einen Beitrag zur Versorgungsforschung in einem bislang nur begrenzt prospektiv untersuchten Setting. Im Folgenden werden die zentralen Ergebnisse im Kontext der bestehenden Literatur eingeordnet und ihre Relevanz für die Weiterentwicklung von Steuerungs- und Versorgungskonzepten diskutiert. Die Ergebnisse zeigen einige deutliche alters- und geschlechtsspezifische Unterschiede im Inanspruchnahmeverhalten und unterstreichen die Notwendigkeit einer zielgruppenspezifischen Patientensteuerung.

### Alters- und Geschlechterverteilung

Unsere Ergebnisse sind konsistent mit früheren Arbeiten, die zeigen konnten, dass die Altersgruppe der über 65-Jährigen einen substanziellen Teil der Notaufnahmepatient:innen darstellt [1]. Mit dem demografischen Wandel ist davon auszugehen, dass dieser Anteil weiter steigt, was erhebliche Konsequenzen für Ressourcenplanung und Steuerung hat [20, 21]. Gleichzeitig zeigen sich deutliche Unterschiede zwischen den Geschlechtern [22]. Frauen sind in den höchsten Altersgruppen überrepräsentiert, was epidemiologisch durch die höhere Lebenserwartung erklärbar ist und aufgrund häufig komplexerer Beschwerdebilder besondere Anforderungen an die Versorgung stellt [3].

### Subjektive Dringlichkeit und Kontaktversuch zur vertragsärztlichen Versorgung

Ein zentrales Ergebnis unserer Studie ist, dass die subjektive Dringlichkeit bei beiden Geschlechtern einer der stärksten Einflussfaktoren für das Aufsuchen einer Notaufnahme war. Dieser Befund stimmt mit Somasundaram et al. [15] und Sitter et al. [23] überein, die ebenfalls die Rolle der subjektiven Risikowahrnehmung als entscheidenden Treiber für Notaufnahmebesuche hervorheben [2]. Auch eine aktuelle Studie aus Deutschland zeigt, dass patientenberichtete Faktoren, insbesondere die subjektiv wahrgenommene Dringlichkeit und bestimmte Beschwerdebilder, mit einer Weiterleitung von Patient:innen aus einer Bereitschaftsdienstpraxis in eine Notaufnahme assoziiert sind [24].

Erkenntnisse aus Skandinavien [8] deuten darauf hin, dass Männer deutlich seltener primärärztliche Angebote (z. B. Hausarztbesuche, Hausbesuche) nutzen als Frauen und häufiger komplexen Versorgungsbedarf aufweisen, wie etwa eine Mehrfachmedikation. Frauen suchten in unserer Studie insgesamt häufiger Kontaktversuche zur vertragsärztlichen Versorgung, bevor sie die Notaufnahme aufsuchten. Dies deckt sich mit Studien, die Frauen eine höhere Compliance im Hinblick auf Vorsorge und Arztkontakte attestieren [9]. Männer hingegen scheinen weniger geneigt, niedrigschwellige Versorgungsangebote zu nutzen, was sich auch in einer stärkeren direkten Inanspruchnahme der Notaufnahme widerspiegeln kann und in der Literatur mit höheren Hospitalisierungs- und Mortalitätsraten in Verbindung gebracht wird [11].

In den bivariaten Analysen zeigte sich zudem, dass Befragte ab 50 Jahren häufiger einen vorherigen vertragsärztlichen Kontaktversuch angaben. Dieser Zusammenhang bestätigte sich jedoch in den multivariablen Regressionsanalysen nicht als unabhängiger Einflussfaktor. Die Ergebnisse deuten darauf hin, dass altersbezogene Unterschiede im vorherigen Kontaktversuch zur vertragsärztlichen Versorgung zumindest teilweise durch andere Faktoren, etwa medizinischer Vorstellungsgrund oder subjektive Dringlichkeit, erklärt werden könnten. Jüngere Patient:innen suchten häufiger direkt die Notaufnahme auf, während ältere Patient:innen eher über ärztliche Zuweisungen kamen. Dieses Muster weist auf eine stärkere Einbindung älterer Menschen in bestehende ambulante Versorgungsstrukturen und eine häufigere vorherige ärztliche Rückkopplung hin. Dieser Befund entspricht internationalen Studien, die belegen, dass Altersunterschiede sowohl in der Inanspruchnahme vertragsärztlicher Versorgung als auch in der Risikowahrnehmung eine zentrale Rolle für die Notfallsteuerung spielen [4], wobei ältere Patient:innen zugleich einen substanziellen und weiter wachsenden Anteil der Notaufnahmepopulation stellen und aufgrund komplexerer Morbiditätsprofile häufiger koordinierte Versorgungswege benötigen [25].

### Medizinischer Vorstellungsgrund

Die Analyse der medizinischen Vorstellungsgründe verdeutlicht bestehende Geschlechterunterschiede. Frauen suchten häufiger wegen Schmerzen, neurologischer oder abdominaler Beschwerden die Notaufnahme auf, während Männer häufiger Verletzungen und urologische Probleme berichteten. Diese Unterschiede entsprechen bekannten Mustern, wonach Frauen häufiger mit unspezifischen oder multiplen Symptomen vorstellig werden [26]. Frauen berichten häufiger Beschwerden wie Übelkeit, Schulter- und Rückenschmerzen sowie atypische Symptome [6], während Männer häufiger typische Leitsymptome angeben, die allgemein mit der vorliegenden Erkrankung assoziiert werden. Diese Unterschiede betreffen die Symptompräsentation und verdeutlichen geschlechtsspezifische Muster beim Aufsuchen der Notaufnahme, ohne dass daraus Unterschiede in der Versorgung abgeleitet werden können.

Demografisch zeigen sich in der vorliegenden Studie signifikante geschlechtsspezifische Unterschiede in der Altersverteilung: Männer waren häufiger in den Altersgruppen < 18 Jahre sowie 50–69 Jahre vertreten, während Frauen überproportional häufig ≥ 70 Jahre waren. Dieses Muster entspricht epidemiologischen Notaufnahmedaten, wonach Frauen aufgrund ihrer höheren Lebenserwartung in höheren Altersgruppen überrepräsentiert sind und häufiger mit chronischen und komplexen Erkrankungen vorstellig werden [1, 3]. Die Altersstruktur beeinflusst damit unmittelbar das beobachtete Beschwerdespektrum der Geschlechter.

Das Alter gilt als zentrale Determinante der Symptompräsentation in der Notaufnahme. Ältere Patient:innen weisen häufiger Multimorbidität, Polypharmazie und funktionelle Einschränkungen auf und präsentieren sich daher typischerweise mit mehreren gleichzeitigen oder unspezifischen Beschwerden statt mit klar abgrenzbaren Leitsymptomen [27, 28]. Unspezifische Präsentationen unterscheiden sich deutlich von den eher ereignisbezogenen oder verletzungsassoziierten medizinischen Verstellungsgründen jüngerer Patient:innen. Vor diesem Hintergrund lassen sich auch unsere Ergebnisse einordnen: Der höhere Anteil älterer Frauen in der Stichprobe dürfte dazu beitragen, dass Schmerzen, abdominale und neurologische Beschwerden häufiger berichtet wurden, da diese Symptomgruppen typischerweise mit chronischen oder multifaktoriellen Erkrankungen im höheren Lebensalter assoziiert sind [28]. Gleichzeitig berichteten Frauen häufiger starke Schmerzen (NRS 7–10), was mit der Literatur zu geschlechtsspezifischen Unterschieden in Schmerzwahrnehmung und -kommunikation übereinstimmt. Gerade im höheren Alter werden Schmerzen zudem häufig als diffus oder unspezifisch beschrieben, was die klinische Einordnung zusätzlich erschwert [5].

Insgesamt verdeutlichen diese Befunde, dass medizinische Vorstellungsgründe, Alter und Geschlecht eng miteinander interagieren. Während jüngere Patient:innen häufiger mit akuten, spezifischen Ereignissen die Notaufnahme aufsuchen, präsentieren sich ältere, insbesondere weibliche Patientinnen häufiger mit komplexen, multimorbiden oder unspezifischen Symptomkonstellationen. Die subjektiv hohe Dringlichkeit bei beiden Geschlechtern unterstreicht dabei, dass nicht allein die klinische Objektivierbarkeit, sondern vor allem die individuelle Symptomwahrnehmung das Inanspruchnahmeverhalten bestimmt.

### Patientensteuerung und digitale Angebote

Rund 2 Drittel der Befragten kannten die Rufnummer des Patientenservices 116117, mit deutlichen Alters- und Geschlechtsunterschieden: Frauen kannten die Rufnummer häufiger, ältere Patientinnen am seltensten. Auch die Online-Informationssuche zu gesundheitlichen Beschwerden zeigte einen klaren Altersgradienten – jüngere Patient:innen gaben deutlich häufiger an, sich vor dem Notaufnahmebesuch online informiert zu haben. Vergleichbare Muster finden sich in der Literatur, wonach die Online-Suche nach Gesundheitsinformationen mit steigendem Alter signifikant abnimmt und Frauen häufiger recherchieren als Männer [29]. Auch die Bereitschaft zur Nutzung digitaler Selbsteinschätzungsangebote zeigt einen deutlichen Altersgradienten. Die entspricht Befunden zur Nutzung von Symptom-Checkern, deren Hauptnutzer:innen deutlich jünger sind [30].

Für integrierte Notfallzentren und strukturierte Ersteinschätzungsverfahren wie SmED bedeutet dies, dass digitale Angebote allein nicht ausreichen, um alle Patient:innengruppen zu erreichen. Sie sollten mit niedrigschwelligen, zielgruppenspezifischen Beratungsangeboten kombiniert werden. Aktuelle Pläne zur strukturierten telefonischen Ersteinschätzung sehen vor, die 116117 als zentralen Zugang zu nutzen, um Patient:innen nach medizinischer Dringlichkeit zu priorisieren und Empfehlungen zu Versorgungswegen zu geben [31]. Insbesondere im Rahmen integrierter Notfallzentren und des geplanten Notfallgesetzes [32] können digitale Symptom-Checker jüngere, digital affine Patient:innen ansprechen, während eine telefonische Ersteinschätzung als niedrigschwelliges und persönliches Angebot für alle Altersgruppen verfügbar bleibt.

### Stärken und Schwächen

Mit der Rekrutierung in 18 bayerischen und 5 Berliner Notaufnahmen deckt die Studie ein breites Spektrum an Versorgungsstrukturen ab, einschließlich urbaner und ländlicher Regionen. Die Studie profitiert von einer insgesamt hohen Fallzahl. Die systematisch rekrutierte Stichprobe repräsentiert einen breiten Querschnitt der Notaufnahmepatient:innen und weist in zentralen Merkmalen wie Alter, Geschlecht und Zugangsweg eine hohe Übereinstimmung mit der Gesamtpopulation des Erhebungszeitraums auf.

Trotz der breiten und systematischen Rekrutierungsstrategie ist ein Selektionsbias durch unterschiedliche Teilnahmebereitschaften nicht auszuschließen. Insbesondere Patient:innen mit Sprachbarrieren, kognitiven Einschränkungen oder ohne begleitende Unterstützung könnten unterrepräsentiert sein, sodass die Stichprobe bestimmte Subgruppen der Notaufnahmepopulation möglicherweise nur eingeschränkt abbildet. Da Patient:innen mit unmittelbarem Behandlungsbedarf (MTS rot/ESI 1) ausgeschlossen wurden, ist die Gruppe der schwerstbetroffenen Patient:innen in der Studie nicht abgebildet. Zudem ist aufgrund der Erhebung auf Basis von Selbstangaben ein potenzieller Bias durch sozial erwünschtes Antwortverhalten nicht auszuschließen, insbesondere hinsichtlich der Selbsteinschätzung von Inanspruchnahmemotiven und der subjektiven Dringlichkeitsbewertung [33].

Eine wichtige Limitation betrifft die Patient:innen unter 18 Jahren. Diese Gruppe ist nicht repräsentativ für pädiatrische Notfallpatient:innen, da keine spezialisierten Kindernotaufnahmen eingeschlossen wurden. Die Ergebnisse dieser Gruppe sind daher mit besonderer Vorsicht zu interpretieren. Die Entscheidung, Patient:innen unter 18 Jahren dennoch einzuschließen, wurde getroffen, um die reale Versorgungsstruktur allgemeiner Notaufnahmen abzubilden. Die zentralen Schlussfolgerungen beziehen sich primär auf die erwachsene Notfallpopulation.

## Fazit

Unsere Ergebnisse zeigen, dass eine geschlechter- und alterssensible Patientensteuerung zwingend erforderlich ist, um Fehlsteuerungen zu reduzieren und die Versorgungsqualität zu verbessern. Dies umfasst erstens die Stärkung niedrigschwelliger Steuerungsinstrumente, wie beispielsweise der 116117 und strukturierter Ersteinschätzungsverfahren, die mit gezielten Aufklärungsmaßnahmen für ältere Patient:innen kombiniert werden sollten. Zweitens sollten geschlechtsspezifische Unterschiede in der Symptompräsentation und in den Nutzungsmustern bei der Gestaltung von Steuerungsinstrumenten systematisch berücksichtigt werden, etwa durch entsprechende Schulungen des Personals und eine Anpassung der Versorgungsangebote. Drittens ist die Integration digitaler Lösungen in hybride Versorgungsmodelle notwendig, um unterschiedliche Alters- und Bildungsschichten adäquat zu erreichen. Viertens bedarf es weiterführender Forschung zum Zusammenwirken von Alter und Geschlecht, insbesondere im Hinblick auf relevante Outcome-Parameter wie Hospitalisierung, Mortalität und Patientenzufriedenheit.

### Research Group – Inanspruchnahmebefragung Berliner und bayerische Notaufnahmen

Dr. Dominik von Stillfried und Johannes Hagelskamp (Zentralinstitut für die kassenärztliche Versorgung in der Bundesrepublik Deutschland), Gökhan Katipoglu und Dr. Christian Pfeiffer (Kassenärztliche Vereinigung Bayerns), Dr. Michael Bayeff-Filloff (RoMed Kliniken), PD Dr. Michael Dommasch (Technische Universität München, Klinikum Rechts der Isar), Prof. Harald Dormann (Klinikum Fürth), Prof. René Hartensuer (Klinikum Aschaffenburg-Alzenau), Dr. Thoms Händl (Klinikum Garmisch-Partenkirchen), Dr. Steffen Herdtle (Krankenhaus Agatharied), Prof. Matthias Klein (Ludwig-Maximilians-Universität Klinikum Campus Großhadern), Dr. Philipp Kellner (Vivantes Klinikum im Friedrichshain), Dr. Marleen Pfeiffer (Wertachkliniken), Dr. Felix Rockmann (Barmherzige Brüder Krankenhaus Regensburg), Prof. Peter Rupp (Klinikum Starnberg), Prof. Rajan Somasundaram (Charité – Universitätsmedizin Berlin), Prof. Georg Osterhoff, Dr. Katja Trommler und Stefan Wollschläger (BG-Unfallkrankenhaus Berlin), Prof. Markus Wehler (Universitätsklinikum Augsburg), Prof. Christian Wrede (Helios Klinikum Berlin-Buch), Prof. Markus Wörnle (Ludwig-Maximilians-Universität Klinikum Campus Innenstadt), PD Dr. Markus Zimmermann (Universitätsklinikum Regensburg).

## Supplementary Information

Onlinematerial: Charakteristika der befragten Patient:innen nach Altersgruppen

## Data Availability

Die während der vorliegenden Studie erzeugten und/oder analysierten Datensätze sind auf begründete Anfrage bei der Korrespondenzperson erhältlich.

## Footnotes

1 MTS (Manchester Triage System) „rot“: sofortige Behandlung erforderlich (akute Lebensgefahr), ESI (Emergency Severity Index) Stufe 1: unmittelbare lebensrettende Intervention notwendig.
