# Supplementary material for: Age and gender aspects in the utilization of emergency departments and their consideration in the management of emergency care
Source: Bundesgesundheitsblatt Gesundheitsforschung Gesundheitsschutz. 2026 Jun 10;69(10):1194–203. [Article in German] doi: 10.1007/s00103-026-04252-4 (PMC13623991; doi:10.1007/s00103-026-04252-4)
Supplement: Supplementary file 1 [file 103_2026_4252_MOESM1_ESM.pdf]

Onlinematerial: Charakteristika der befragten Patient:innen nach Altersgruppen

	Unter 18 Jahre		18-29 Jahre		30- 39 Jahre		40-49 Jahre		50-59 Jahre		60-69 Jahre		70-79 Jahre		Ab 80 Jahre		
	Anzahl	Anteil in %	Anzahl	Anteil in %	Anzahl	Anteil in %	Anzahl	Anteil in %	Anzahl	Anteil in %	Anzahl	Anteil in %	Anzahl	Anteil in %	Anzahl	Anteil in %	p Wert
**Geschlecht (n=9.818)**	669	6,80	1.418	14,40	1.297	13,20	1.194	12,20	1.347	13,70	1.350	13,80	1.223	12,50	1.320	13,40	
Weiblich	275	5,60	732	15,00	633	13,00	564	11,60	622	12,80	634	13,00	650	13,30	762	15,60	
Männlich	394	8,00	686	13,90	664	13,40	630	12,70	725	14,70	716	14,50	573	11,60	558	11,30	*<0,001*
**Bildungstand nach ISCED** *(n=8.973)*	432	4,80	1350	15,00	1245	13,90	1112	12,40	1269	14,10	1272	14,20	1122	12,50	1171	13,10	
Niedrig	314	26,20	311	25,90	95	7,90	83	6,90	80	6,70	74	6,20	99	8,30	144	12,00	
Mittel	48	1,00	703	15,30	566	12,40	538	11,70	650	14,20	732	16,00	642	14,00	702	15,30	*<0,001*
Hoch	70	2,20	336	10,50	584	18,30	491	15,40	539	16,90	466	14,60	381	11,90	325	10,20	
**Migration** *(n=9.762)*	666	6,80	1.417	14,50	1.298	13,30	1.185	12,10	1.339	13,70	1.337	13,70	1.219	12,50	1.301	13,30	
Kein Migrationshintergrund	538	7,20	965	12,90	863	11,50	799	10,70	1.027	13,70	1.116	14,90	1.051	14,00	1.141	15,20	*<0,001*
Migrationshintergrund	128	5,70	452	20,00	435	19,20	386	17,10	312	13,80	221	9,80	168	7,40	160	7,10	
**Zugangsweg** *(n=9.670)*	658	6,80	1.395	14,40	1.284	13,30	1.176	12,20	1.316	13,60	1.333	13,80	1.210	12,50	1.298	13,40	
Selbstständig	603	8,60	1.177	16,70	1.088	15,50	959	13,60	1.014	14,40	945	13,40	730	10,40	525	7,50	*<0,001*
Rettungsdienst / Krankentransport	55	2,10	218	8,30	196	7,50	217	8,30	302	11,50	388	14,80	480	18,30	773	29,40	
**Medizinischer Vorstellungsgrund** *(n= 9.875; Mehrfachantworten)*																	
Allgemeine Beschwerden	27	4,00	83	5,80	70	5,40	71	5,90	87	6,40	77	5,70	99	8,00	131	9,90	*<0,001*
Schmerzen	202	30,10	434	30,20	365	27,90	364	30,40	401	29,70	377	27,80	304	24,70	338	25,50	*<0,001*
Verletzung	421	62,80	435	30,30	328	25,10	275	22,90	282	20,90	245	18,10	206	16,70	310	23,40	*<0,001*
Herz-Kreislauf Beschwerden / Atembeschwerden	11	1,60	132	9,20	141	10,80	158	13,20	235	17,40	297	21,90	319	25,90	321	24,20	0,060
Bauchbeschwerden	32	4,80	152	10,60	154	11,80	139	11,60	151	11,20	151	11,10	128	10,40	117	8,80	0,621
Neurologische Beschwerden / psychiatrische Beschwerden	3	0,40	129	9,00	94	7,20	100	8,30	123	9,10	115	8,50	100	8,10	126	9,50	*<0,001*
Beschwerden auf der Haut	15	2,20	40	2,80	52	4,00	37	3,10	23	1,70	27	2,00	31	2,50	18	1,40	*0,002*
Beschwerden im Hals-Nasen Ohren Bereich	28	4,20	90	6,30	71	5,40	43	3,60	37	2,70	35	2,60	34	2,80	17	1,30	*<0,001*
Urologische Beschwerden	4	0,60	48	3,30	43	3,30	36	3,00	52	3,80	57	4,20	70	5,70	60	4,50	
Gynäkologische Beschwerden	4	0,60	60	4,20	62	4,70	20	1,70	6	0,40	5	0,40	4	0,30	3	0,20	
Augenprobleme	13	1,90	33	2,30	28	2,10	29	2,40	38	2,80	36	2,70	27	2,20	15	1,10	0,274
**NRS-Schmerzabfrage** *(n=9.986)*	536	8,00	1.088	16,30	980	14,70	887	13,30	922	13,80	856	12,80	690	10,30	730	10,90	
Gering (1-3)	74	7,40	165	16,50	159	15,90	136	13,60	154	15,40	117	11,70	93	9,30	99	9,90	
Mittel (4-6)	236	9,90	406	17,00	346	14,50	294	12,30	303	12,70	305	12,80	257	10,80	236	9,90	*<0,001*
Stark (7-10)	226	6,80	517	15,60	475	14,40	457	13,80	465	14,10	434	13,10	340	10,30	395	11,90	
**Subjektive Dringlichkeit** *(n=9.592)*	654	6,80	1.395	14,50	1.270	13,20	1.168	12,20	1.319	13,80	1.319	13,80	1.202	12,50	1.265	13,20	
Nicht dringlich	14	10,40	24	17,80	19	14,10	19	14,10	14	10,40	14	10,40	14	10,40	17	12,60	*<0,001*
Weniger dringlich	95	9,20	227	21,90	157	15,20	106	10,20	144	13,90	107	10,30	99	9,60	101	9,70	

Dringlich	335	8,50	616	15,60	547	13,80	498	12,60	516	13,10	513	13,00	449	11,40	476	12,10
Sehr dringlich	156	5,40	351	12,20	359	12,50	348	12,10	406	14,10	419	14,50	412	14,30	429	14,90
Notfall	54	3,40	177	11,10	188	11,80	197	12,40	239	15,00	266	16,70	228	14,30	242	15,20

Legende: ISCED: International Standard Classification of Education; NRS: Numerische Rating-Skala
